# Supplementary material for: Measurement of Exhaled Volatile Organic Compounds as a Biomarker for Personalised Medicine: Assessment of Short-Term Repeatability in Severe Asthma
Source: J Pers Med. 2022 Oct 2;12(10):1635. doi: 10.3390/jpm12101635 (PMC9604907; doi:10.3390/jpm12101635)
Supplement: Supplementary file 1 [file jpm-12-01635-s001.zip › jpm-1889124-supplementary.pdf]

Supplementary Table S1

Principal Component Loadings for the first 10 Principal Components. Principal Components (PC)

ID	PC1	PC2	PC3	PC4	PC5	PC6	PC7	PC8	PC9	PC10
Butane, 2-methyl-	-0.07926	0.009723	0.113709	0.03363	-0.14753	0.066121	-0.06074	0.306388	-0.22803	0.408603
Sevoflurane	-0.03944	0.096948	0.047568	0.017491	0.177325	-0.10126	-0.06109	0.089542	-0.12718	-0.11868
Trichloromonofluoromethane	-0.07826	0.135891	-0.07545	-0.03723	0.119888	-0.18485	-0.16184	0.070488	-0.01489	0.422441
Pentane	-0.06622	0.056339	0.132765	-0.05799	-0.03544	-0.04205	-0.16697	0.215808	-0.09865	0.235983
Dimethyl selenide	0.08843	0.063819	0.259308	-0.13825	-0.11966	-0.06366	-0.06445	-0.10034	-0.24362	-0.11449
Methacrolein	-0.03398	0.029706	0.096891	-0.24538	-0.06273	-0.14844	-0.02906	-0.08444	-0.13214	-0.0685
Furan, 2-methyl-	-0.02433	0.020357	0.116907	-0.28238	-0.09206	-0.20648	-0.03101	-0.05565	-0.03128	-0.03945
2-Butanone	-0.03511	0.025004	0.059132	-0.05413	0.002399	-0.04601	-0.05478	0.000954	-0.00938	0.022043
1,3,5-Trifluorobenzene	-0.07933	0.102564	-0.15035	-0.15579	0.009297	-0.08676	-0.11944	0.040786	0.081934	-0.0169
Trichloromethane	-0.08699	0.079835	0.078352	-0.07649	0.032394	-0.10613	-0.3272	0.252132	0.189509	0.059116
Benzene, 1,2-difluoro-4-(trifluoromethyl)-	-0.04536	0.053572	-0.09979	-0.14686	-0.00319	-0.06453	-0.10951	0.028604	0.041106	-0.06042
Carbon Tetrachloride	-0.09295	0.291258	-0.08948	-0.00077	0.116463	-0.15301	-0.12837	-0.07687	-0.30203	0.055317
1,2,3-Trifluoro-4-trifluoromethylbenzene	-0.03363	0.088939	-0.07943	-0.09861	0.002558	-0.08424	-0.08506	0.057149	0.031959	0.002142
2-Pentanone	-0.04476	-0.02673	0.027708	0.030114	-0.01532	0.08415	0.014815	0.193095	-0.03176	-0.10189

1,4-Dioxane	-0.07846	0.030664	0.023251	-0.19601	-0.06573	0.032953	-0.13144	0.145997	-0.13398	-0.10947
1-Propene, 1-(methylthio)-, (Z)-	0.086834	0.148704	0.333556	0.069036	-0.19246	-0.11907	0.036657	-0.13633	-0.10523	0.044314
1-Propene, 1-(methylthio)-, (E)-	0.104573	0.132965	0.38654	0.090414	-0.17715	-0.11472	0.035994	-0.14981	-0.07659	0.066779
Octane	0.013677	0.079701	-0.04179	0.011512	0.120224	0.101763	0.238478	0.00333	-0.09694	0.123582
Tetrachloroethylene	-0.03359	-0.05944	0.120964	-0.25924	0.091543	0.113873	0.237695	0.203805	0.375281	0.123236
2,3,4-Trifluorobenzoic acid, 4-nitrophenyl ester	-0.01187	0.116834	-0.1104	-0.05706	0.025827	0.036453	-0.09533	-0.01532	0.030661	-0.08292
Hexanal	-0.06216	0.326353	0.028936	-0.03094	-0.02898	0.157488	-0.00414	0.11767	0.045995	-0.13264
1,1,3,3-Tetramethyl-1,3-bis[(2Z)-pent-2-en-1-yloxy]disiloxane	-0.01203	0.19898	-0.09367	0.098975	0.065862	0.054473	0.247995	0.041656	-0.11028	0.054676
Cyclohexane, 1,3,5-trimethyl-	0.056186	-0.09801	0.236713	-0.05824	0.284991	-0.18257	0.006889	-0.0144	0.0504	-0.02177
Heptane, 2,3-dimethyl-	-0.02758	0.004062	0.155133	0.09534	0.284509	0.089762	0.207532	-0.10797	-0.05772	0.216686
Octane, 2-methyl-	0.001542	-0.01287	0.17985	-0.03063	0.264399	-0.15736	0.078525	0.078117	-0.00373	0.032987
p-Xylene	-0.05947	-0.03175	0.056693	-0.02477	0.209709	0.042463	-0.03732	0.142415	-0.1317	-0.09856
Nonane	0.035062	0.076328	0.279138	-0.05612	0.215761	-0.1113	0.107653	0.162563	0.199645	-0.07183
4-Heptanone	-0.00193	-0.07239	-0.01054	0.023481	0.036702	0.084298	0.056337	0.227921	-0.07643	-0.07476
o-Xylene	-0.05301	-0.02178	0.034017	-0.00394	0.210001	0.003109	-0.08006	0.16597	-0.10994	-0.13271

3-Methylcyclopentyl acetate	-0.09826	0.508896	-0.16518	0.026833	-0.05254	0.141114	0.004351	-0.25176	0.200014	-0.013
Heptanal	-0.04119	0.257495	0.060101	-0.13137	-0.09412	0.125174	0.19836	0.188629	-0.02422	0.007515
Bicyclo[3.1.0]hex-2-ene, 4-methyl-1-(1-methylethyl)-	0.306851	0.155725	-0.07826	0.017456	0.059506	-0.05535	-0.1061	0.063042	0.069441	0.085971
?	-0.00804	0.014618	-0.08923	-0.26934	-0.03161	-0.00528	-0.16976	-0.06115	-0.01247	0.06312
Camphene	0.2771	0.15573	0.199088	0.043658	-0.10842	0.131919	-0.04734	-0.07999	0.083361	0.158339
.beta.-Pinene	0.246921	0.216226	-0.18323	0.100406	0.151296	-0.05473	-0.02439	0.08179	0.047066	0.111742
Heptane, 2,2,4,6,6-pentamethyl-	0.044112	0.209171	0.047627	-0.23658	0.095661	-0.21132	0.188729	-0.12724	0.048798	-0.15256
.beta.-Myrcene	0.2847	-0.00457	-0.08094	-0.06249	0.087352	-0.02129	0.026866	0.136832	-0.09049	-0.16547
Decane	-0.06461	0.067735	0.07639	-0.07462	0.384652	0.025014	0.05511	-0.25928	0.088586	0.076927
3-Carene	0.137075	0.172371	0.033168	-0.02802	0.047059	-0.0048	-0.16444	-0.01061	-0.13406	-0.19014
1,3-Cyclohexadiene, 1-methyl-4-(1-methylethyl)-	0.353069	-0.02419	-0.05966	-0.00105	0.010768	0.072761	-0.05838	0.043418	-0.1147	-0.05644
Octanal	-0.06754	0.23593	0.039494	-0.11438	-0.07165	0.289939	0.108997	0.189328	0.003799	-0.14024
D-Limonene	0.116352	-0.0673	-0.13087	-0.05131	0.229301	-0.01253	-0.19851	-0.00251	-0.08439	-0.10525
p-Cymene	0.316522	0.00072	0.028506	0.024321	0.13818	0.163118	-0.16304	-0.01721	0.133606	0.020679
Eucalyptol	0.163398	-0.05529	0.064295	-0.16083	-0.15631	0.135969	-0.22848	0.045606	0.198788	0.117892

.beta.-Ocimene	0.153439	0.015468	0.199102	0.012778	-0.09337	0.087101	-0.00502	0.012589	0.079292	-0.13951
.gamma.-Terpinene	0.255654	0.079303	-0.01858	0.121971	0.12537	0.067257	-0.0469	0.03955	-0.00734	0.026562
4-Cyanocyclohexene	-0.0405	0.028341	0.132199	-0.05469	0.10432	0.243671	-0.08976	0.054135	0.061164	0.189061
Octane, 1-chloro-	-0.04937	0.058553	0.079084	-0.05425	0.0183	0.266757	0.034205	0.094956	-0.08795	-0.16963
Cyclohexene, 1-methyl-4-(1-methylethylidene)-	0.243282	-0.02645	0.065519	0.102182	0.039444	0.119771	-0.10766	0.006759	-0.12669	-0.00405
Undecane	-0.0443	-0.00603	0.126642	-0.16876	0.139069	0.089044	-0.10309	-0.02824	0.044908	-0.0822
3-Octanol, 3,7-dimethyl-	0.010617	0.062792	-0.05469	0.031485	0.068923	0.014349	0.170108	0.172176	-0.37881	0.032168
Undecane, 3-methyl-	-0.04651	-0.16462	0.097693	-0.23282	0.076677	0.385266	-0.08299	-0.1913	-0.16461	-0.03885
l-Menthone	0.211559	-0.08405	-0.1236	-0.28879	-0.09143	-0.06943	0.23996	0.022025	-0.10313	0.104095
Dodecane	-0.04994	-0.03687	-0.06515	-0.25417	0.128177	0.283167	-0.09447	-0.3199	-0.19671	0.2432
Cyclohexanol, 5-methyl-2-(1-methylethyl)-, (1.alpha.,2.alpha.,5.beta.)-	0.178743	-0.03037	-0.16438	-0.2602	-0.04286	-0.07038	0.18957	0.038306	-0.01113	0.023311
Cyclohexanone, 5-methyl-2-(1-methylethyl)-, trans-	0.216861	-0.04904	-0.12412	-0.27753	-0.08419	-0.08247	0.206004	0.052114	-0.01896	0.162814
